# Identification of Key Volatile Organic Compounds Released by Gastric Tissues as Potential Non-Invasive Biomarkers for Gastric Cancer

**DOI:** 10.3390/diagnostics13030335

**Published:** 2023-01-17

**Authors:** Paweł Mochalski, Marcis Leja, Daria Ślefarska-Wolak, Linda Mezmale, Veronika Patsko, Clemens Ager, Agnieszka Królicka, Chris A. Mayhew, Gidi Shani, Hossam Haick

**Affiliations:** 1 Institute of Chemistry, Jan Kochanowski University of Kielce, PL-25406 Kielce, Poland; 2 Institute for Breath Research, University of Innsbruck, A-6850 Dornbirn, Austria; 3 Institute of Clinical and Preventive Medicine, Faculty of Medicine, University of Latvia, LV-1586 Riga, Latvia; 4 Digestive Diseases Centre GASTRO, LV-1586 Riga, Latvia; 5 Riga East University Hospital, LV-1586 Riga, Latvia; 6 National Cancer Institute of Ukraine, 03022 Kyiv, Ukraine; 7 Department of Building Materials Technology, Faculty of Materials Science and Ceramics, AGH University of Science and Technology, Mickiewicza 30, PL-30059 Krakow, Poland; 8 Department of Chemical Engineering, Russel Berrie Nanotechnology Institute, Technicon—Israel Institute of Technology, Haifa 3200003, Israel

**Keywords:** gastric cancer, biomarker, gastric tissue, volatile organic compound, GC-MS

## Abstract

Background: Volatilomics is a powerful tool capable of providing novel biomarkers for medical diagnosis and therapy monitoring. The objective of this study is to identify potential volatile biomarkers of gastric cancer. Methods: The volatilomic signatures of gastric tissues obtained from two distinct populations were investigated using gas chromatography with mass spectrometric detection. Results: Amongst the volatiles emitted, nineteen showed differences in their headspace concentrations above the normal and cancer tissues in at least one population of patients. Headspace levels of seven compounds (hexanal, nonanal, cyclohexanone, 2-nonanone, pyrrole, pyridine, and phenol) were significantly higher above the cancer tissue, whereas eleven volatiles (ethyl acetate, acetoin, 2,3-butanedione, 3-methyl-1-butanol, 2-pentanone, γ-butyrolactone, DL-limonene, benzaldehyde, 2-methyl-1-propanol, benzonitrile, and 3-methyl-butanal) were higher above the non-cancerous tissue. One compound, isoprene, exhibited contradictory alterations in both cohorts. Five compounds, pyridine, ethyl acetate, acetoin, 2,3-butanedione, and 3-methyl-1-butanol, showed consistent cancer-related changes in both populations. Conclusions: Pyridine is found to be the most promising biomarker candidate for detecting gastric cancer. The difference in the volatilomic signatures can be explained by cancer-related changes in the activity of certain enzymes, or pathways. The results of this study confirm that the chemical fingerprint formed by volatiles in gastric tissue is altered by gastric cancer.

## 1. Introduction

A number of recent studies have provided robust evidence that volatilomics is effective in identifying health issues and diagnosing diseases [1,2]. The term “*volatilome*” is used to represent a subset of the metabolome, comprising volatile organic compounds (VOCs) of various origins from within the human organism. As a whole, they provide specific biochemical signatures that contain information on various normal and abnormal metabolic processes occurring in the body. Pathological processes such as oxidative stress, changes in enzyme activity, carbohydrate metabolism, lipid metabolism, modifications of proteins, or activation of genes alter these signatures [3,4,5]. Volatilomics targets these changes in different bodily excretions, such as breath, skin emanations, urine, saliva, or sweat to monitor or screen for various diseases, including cancer. Importantly, VOC biomarkers are expected to occur in the early development of a disease. If so, volatile biomarkers can potentially provide an even earlier detection of recurrence or progression than conventional markers. The unique feature of this concept is that the information on the processes occurring in the human organism is obtained non-invasively via analysis of volatiles emitted or secreted by the human body into its surrounding environment. Thus, VOC sampling is well tolerated by patients and can be performed even at home as often as required. Being non-invasive, this VOC approach can lead to novel, fast, accessible, and safe diagnostic tests. Moreover, volatilomic signatures can be analysed using simple and cheap analysers based on miniature sensors, such as chemical sensors [1,6,7] or biosensors [8]. Therefore, volatilomic signatures of disease are the subjects of intense research associated with biomarker discovery.

Gastric cancer is the fifth most diagnosed malignancy worldwide with over 1 million estimated new cases per year, and is the third most common cause of cancer death globally [9]. Mortality from gastric cancer is high. For example, there were approximately 784,000 deaths globally in 2018. In its early stages, symptoms are frequently lacking, resulting in an often late and incurable diagnosis and a 5-year-survival rate of 32%. Since an early diagnosis is crucial for a patient’s survival, a rapid and non-invasive screening system is of the highest importance.

Over the last decade, volatilomics has been employed to detect and classify gastric cancer using volatiles contained in human exhaled breath, yielding promising results [10,11]. Principally, there are two distinct approaches to capture VOC signatures in exhaled breath: (1) molecularly detailed gas chromatography coupled to mass-spectroscopy (GC-MS), and (2) sensors coupled to pattern recognition algorithms. Sensor technologies recognise so-called ‘breath fingerprints’ (a.k.a., ‘*breathprints*’) rather than identifying the volatiles and apply them in searching for a particular disease. Instruments based on these technologies have been demonstrated to discriminate gastric cancer patients from other patients and controls with a sensitivity and specificity ranging from 0.67 to 1.0 and from 0.71 to 0.98, respectively [10]. A particularly fascinating example of this approach is a study by Nakhleh et al. [12] reporting the non-invasive diagnosis and classification of seventeen diseases, including gastric cancer, from exhaled breath using nanomaterial-based sensors. Although the chemical sensor-based technologies show considerable promise for use in volatilomics, an unresolved issue is their very low selectivity, i.e., their inability to identify substances causing the breathprints. To develop sensor breath tests, the VOCs forming the breathprints need to be identified with a high level of confidence, which can be used to optimise the sensor-based technologies and subsequently support the optimisation of sensor-based tests aiming at the respiratory markers. Furthermore, the identification of the VOCs provides important information to help better understand the metabolic processes resulting in their production. The chemical identity of potential markers of gastric cancer can be revealed by highly selective techniques such as GC-MS analysing the VOCs contained in different bodily fluids and tissues. In this context, tissue samples are particularly attractive, as they offer direct access to cancer cells, which provides the possibility to directly pinpoint cancer-related alterations in their metabolism [13,14,15].

The ambition of this study is to identify the potential volatile biomarkers of gastric cancer. Specifically, this study centres upon the volatilomic signatures of gastric tissues to capture alterations in their metabolism induced by cancer. This embraces the identification of VOCs released by cancer and non-cancerous tissue and the identification of the volatile species being expressed at different levels in order to provide an alternative source of information on gastric cancer-related biomarkers. To improve the reliability of information, two distinct geographic populations of gastric cancer patients, namely, in Latvia and Ukraine, have been included in this study. The volatilomic signatures of tissue samples were investigated using the headspace solid phase microextraction (HS-SPME), followed by GC-MS.

## 2. Materials and Methods

### 2.1. Chemicals and Standards

All standard mixtures were produced using high-purity liquid substances. Reference chemicals with stated purities of 95–99.9% were purchased from Merck (Austria) and Fluka (Switzerland). Standards were prepared by injecting and evaporating several microlitres of the liquid compound into evacuated and heated 1 L glass bulbs (Supelco, Bellefonte, ON, Canada). Different calibration concentrations were obtained by transferring appropriate volumes from the bulb mixtures into Tedlar bags (SKC Inc., Eighty Four, PA, USA) that were pre-filled with purified and humidified air (relative humidity 100% at 34 °C). Ultimately, gas mixtures with VOC volume fractions ranging from 0.07 to 160 parts per billion by volume (ppb) were used for calibration. The calibration curves relied on using seven distinct and independent concentration levels.

### 2.2. Subjects and Tissues Sampling

A cohort of 94 patients diagnosed with gastric cancer was recruited, all of whom were scheduled for elective gastric surgery. These patients were recruited in two clinical centres: the National Cancer Institute of Ukraine (UNCI) and the Riga East University Hospital, Latvian Oncology Centre (LOC). Details on the patient numbers, sex, and age range and median from these two centres are summarised in Table 1.

The tissue sampling, sample storage, and transport followed the standardized protocols outlined in detail elsewhere [15]. Therefore, only a short description will be provided here. Tissue samples were taken during gastric surgery; cancerous tissue as well as normal tissue without malignant infiltration were resected from each patient. The healthy and malignant tissues were evaluated by gross examination, followed by histological processing. Approximately 100 mg of each tissue were provided for the analytical measurements. These samples were transferred into 2 mL amber vials, snap-frozen in liquid nitrogen, and placed in a −80 °C freezer for storage with a maximal storage time of 6 weeks. Transportation was in the frozen state with samples kept on dry ice.

### 2.3. HS-SPME Sampling Protocol

Volatiles released by tissue samples were extracted using headspace solid phase microextraction (HS-SPME). Shortly before analysis, samples were thawed at 40 °C for 5 min and 100 mg (±10%) of a frozen tissue sample were transferred into a headspace vial (20 mL, Gerstel, Mülheim an der Ruhr, Germany). Next, the vial was rinsed with high purity nitrogen 99.9999% (approximately 100 mL at the flow rate of 30–40 mL × min^−1^) and crimped with 1.3 mm butyl/PTFE septa.

HS-SPME was carried out automatically using a multipurpose sampler MPS (Gerstel, Mülheim an der Ruhr, Germany). Extraction was achieved by inserting a 75 μm Carboxen-PDMS SPME fibre (Supelco, Bellefonte, ON, Canada) into the vials and exposing it to the headspace gas for 50 min. Subsequently, the fibre was introduced into the inlet of the gas chromatograph where the volatiles of interest were thermally desorbed at 290 °C in a splitless mode (1 min). The fibre was conditioned at 290 °C for 5 min prior to each analysis. In parallel with each pair of tissue samples, one blank sample containing nitrogen was analysed using the same protocol to identify possible contaminants stemming from sources other than the tissues. The resulting concentration levels were subtracted (where applicable) from the respective values in the associated tissue samples. Samples were analysed in batches on a daily randomized regime.

### 2.4. GC-MS Analysis

GC-MS analysis relied on an Agilent 8890/7079B GC-MS (Agilent, Santa Clara, CA, USA). The GC injector was equipped with an inert SPME liner (inner diameter 0.75 mm, Supelco, Canada) and operated in the splitless mode (0.75 min), followed by split mode with a ratio 1:50. Extracted compounds were separated using an Rxi-624Sil MS column (30 m × 0.32 mm, layer thickness 1.8 μm, Restek, Bellefonte, PA, USA) operated in constant helium flow at 1.4 mL min^−1^. The column temperature programme was as follows: 37 °C for 12 min, followed by 5 °C min^−1^ up to 150 °C, then 10 °C min^−1^ up to 290 °C, and finally remaining at 290 °C for 8 min. The untargeted VOC analysis relied on the mass spectrometer working in a SCAN mode with the associated m/z ranging from 20 up to 250. The peak integration was based on extracted m/z ratio chromatograms, and such an approach allowed for a separation of the majority of peaks of interest from their neighbours. The quadrupole, ion source, and transfer line were kept at 150 °C, 230 °C and 280 °C, respectively.

VOC identification was performed using a two-step process. First, the spectrum of a peak was checked against the NIST mass spectral library database. Next, the NIST identification was confirmed by comparing the retention times of peaks of interest with retention times obtained for reference standards prepared as outlined above.

## 3. Results

### 3.1. Validation Parameters

The validation parameters are provided in Table 2. Limits of detection (LODs) were calculated, using five consecutive blank signals [10]. The limit of quantification (LOQ) was defined as 3 × LOD. The LOD ranges from 0.02 to 1.17 ppb. Relative standard deviations (RSDs) varied from 5 to 19%. These values were considered adequate for the purposes of this study. The instrument response was found to be linear within the investigated concentration ranges, with coefficients of determination ranging from 0.989 to 0.998.

### 3.2. Volatilomic Signatures of Gastric Cancer and Non-Cancerous Tissues

**Figure 1:**
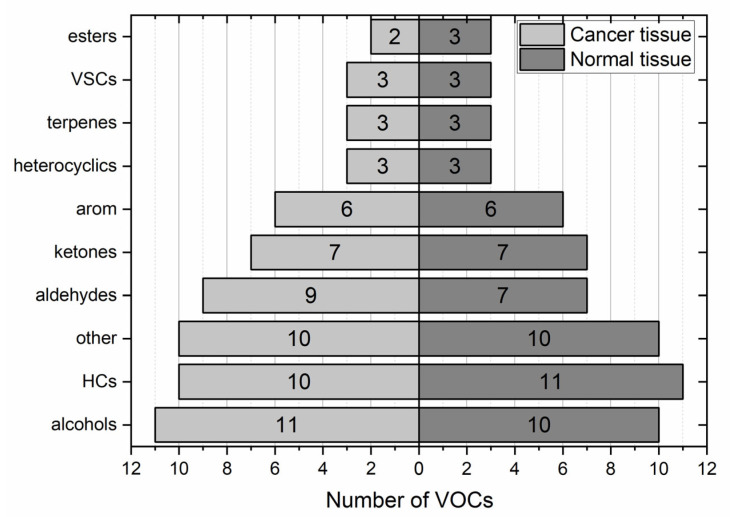
Relative distribution of VOCs with occurrence > 20% according to the chemical classes in all cancerous tissue samples (**left** panel) and all normal tissue samples (**right** panel).

Collectively, a total of 235 compounds were found in the headspace of the tissue samples provided by both centres. Amongst these, 198 VOCs were identified in gastric cancer tissues and 199 in non-cancerous samples. A total of 165 volatiles were found in both types of samples. The majority of these species exhibited very low incidence. Only 32% of VOCs exhibited incidence higher than 20%. Excluding some obvious hospital environment-related species and their metabolites (e.g., sevoflurane, desflurane, propofol, or hexafluoroisopropanol), ten volatiles (2-butanone, benzaldehyde, 2-pentanone, n-pentane, toluene, acetone, carbon disulfide, ethanol, ethyl acetate, and benzonitrile) occurred in at least 80% of both types of samples and thereby can be considered as omnipresent. Moreover, pyridine and 1-propanol exhibited an incidence higher than 80% in tumour tissues, whereas in non-cancerous samples, this threshold was exceeded by acetoin and 2,3 butanedione. The distribution of VOCs with incidence above 20% according to their chemical classes is presented in Figure 1.

It is not surprising that the distribution of the VOCs according to their chemical classes in both types of samples is similar. Each pair of tissue samples stems from the same individual and shares the same exposure to environmental, dietary, disease, or treatment-related VOCs, for examples. The predominant chemical classes in both tissues are hydrocarbons (17%) and alcohols (16%). These are followed by aldehydes (14% in cancer tissues vs. 11% in normal tissue), ketones (both 11%) and aromatics (both 10%). The signal of several compounds (e.g., ethanol, acetone, and 1-propanol) exceeded the dynamic range of the MS detector. Consequently, it was not possible to compare the emission of these metabolites between respective tissue samples.

**Figure 2:**
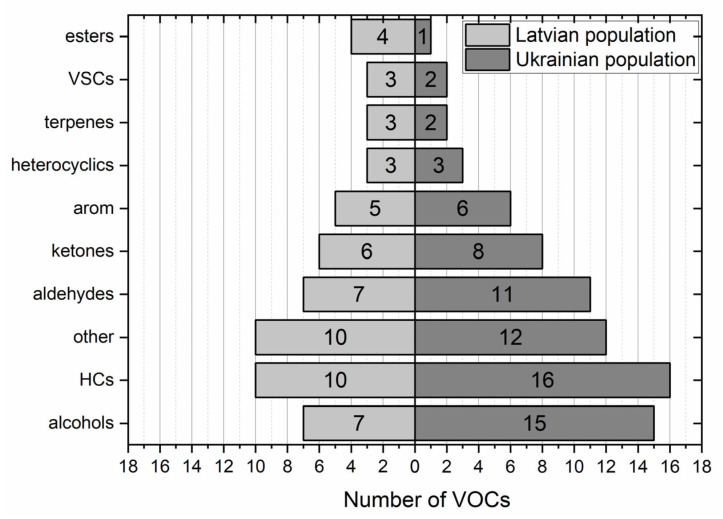
Relative distribution of VOCs with occurrence > 20% according to the chemical classes in cancer tissue of Latvian (**left** panel) and Ukrainian (**right** panel) cohorts of patients.

Both populations under study exhibited some differences in the volatilomic signatures. For instance, in samples obtained from the Ukrainian cohort, 76 VOCs exhibited occurrence exceeding 20%, whereas in the Latvian group of patients, this number amounted to 58. Interestingly, there were differences in the distribution of compounds according to their chemical classes between both involved populations. Considering the cancer tissue, they were manifested mainly by the higher number of detected alcohols (15 vs. 7) and hydrocarbons (16 vs. 10), as depicted in Figure 2. It is difficult to indicate the reason for this disparity; however, some local factors such as the exposure to VOCs coming from the environment (e.g., found in the hospital or from air pollution), diet, microbiota, or treatment-related VOCs might play a considerable role. Nevertheless, these results provide evidence that the human volatilome can be strongly affected by local factors, which results in the, significant variability between different populations can occur.

### 3.3. Differences between the Volatilomic Signatures of Healthy and Cancerous Gastric Tissues

A Wilcoxon signed rank test was used to compare the emissions of VOCs from cancer and non-cancerous tissues, and a *p* < 0.05 was taken as being significant. For this purpose, only compounds with incidence above 50% were taken into consideration. The list of compounds exhibiting a difference in emission between normal and cancerous tissues is presented in Table 3.

Amongst the volatiles detected, 19 showed consistent differences in their headspace concentrations above samples under study. Of these, 11 compounds were found to have reduced emissions from the cancer tissue, and hence the other eight showed reduced release from the healthy one. Four compounds, ethyl acetate, acetoin, 2,3-butanedione, and 3-methyl-1-butanol, exhibited a downregulated production in tumour tissue in both populations. Furthermore, three other volatiles, 2-pentanone, γ-butyrolactone, and DL-limonene, were found to be released in lower amounts only from cancer tissue obtained from Latvian patients. In comparison, four VOCs, benzaldehyde, 2-methyl-1-propanol, benzonitrile, and 3-methyl-butanal, showed analogous alteration only in Ukrainian subjects. The largest drop in the emission by tumour tissue was found for alcohols. Thus, for example, the production of 2-methyl-1-propanol in cancer tissue samples was reduced by a factor of 3.4, whereas 3-methyl-1-butanol exhibited 3 to 7.7 fold downregulated production in these type of samples.

Both populations of interest also showed significant differences with respect to the cancer-related changes leading to the upregulation of VOC production. The species emitted in higher amounts by cancerous tissue comprised two aldehydes (hexanal and nonanal), two ketones (cyclohexanone and 2-nonanone), two heterocyclic VOCs (pyrrole and pyridine), and phenol. One compound, isoprene, exhibited contradictory alterations in emission in both cohorts. In the Latvian cohort, isoprene showed lowered emission from cancer tissue samples, whereas the opposite alteration took place in the Ukrainian population. Only one species, pyridine, exhibited elevated emission from cancer tissues as compared to the normal ones in both populations of patients. Four VOCs, pyrrole, phenol, cyclohexanol, and hexanal, were released in higher amounts by cancer tissues in the Latvian population only, whereas nonanal and 2-nonanol were found to be elevated in the headspace of the tumour samples obtained from the Ukrainian patients. The highest cancer-related change in the emission was noted for pyridine. The cancerous tissue exhibited 2.2 (Latvian patients) and 6.3 (Ukrainian patients) fold upregulated production of this VOC.

An effort was made to quantify all compounds showing the differences in emission. Sixteen compounds were quantified using the aforementioned procedures. Three VOCs, benzaldehyde, acetoin, and phenol, were not quantified due to problems related to the preparation of reliable reference mixtures. Instead, their levels were assessed only based on peak areas. The median headspace concentrations ranged from 0.2 to 163 ppb. However, the majority of quantified species had emissions below 10 ppb. The highest median levels were noted for pyridine (87 and 27 ppb from cancer and normal tissues, respectively). The observed concentrations of volatiles of interest over the tissue samples are provided in Table 4.

**Figure 3:**
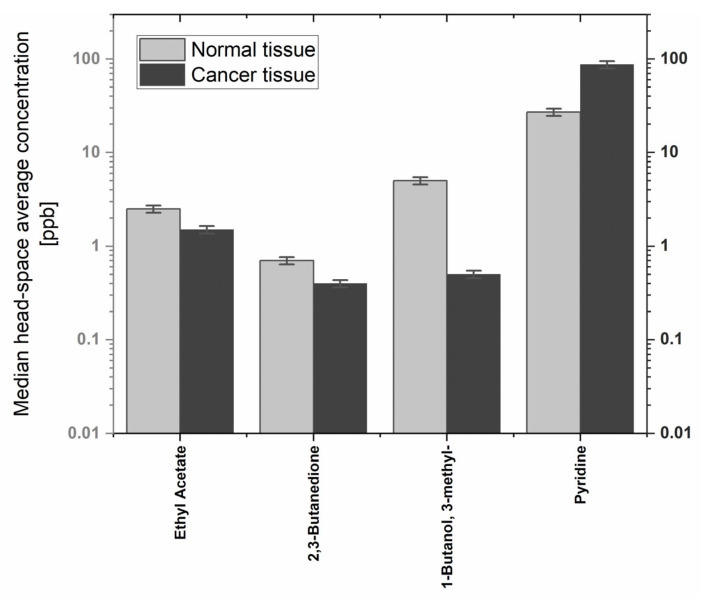
VOCs exhibiting differences in emission between normal and cancer tissues in both cohorts of patients.

The comparison of levels of VOCs showing consistent alterations in both populations of interest is shown in Figure 3.

## 4. Discussion

Although the underlying metabolic routes leading to the production of VOCs of interest are in many cases uncertain, a number of pathways could be responsible for their emission. Primary alcohols such as 2-methyl-1-butanol or 3-methyl-1-butanol may result from the activity of alcohol dehydrogenases (ADHs) that can reversibly reduce aldehydes to alcohols [16]. Thus, 2-methyl-1-propanol can stem from the reduction of 2-methylpropanal, whereas 3-methyl-1-butanol can be the product of the 3-methyl-1-butanol metabolism. Indeed, both 3-methylbutanal and 2-methylpropanal were found in the headspace of the tissue samples. Within this study, the emission of alcohols was significantly lower from cancer tissue in comparison to the normal tissue. This alteration may be attributed to the overexpression of aldehyde dehydrogenases (ALDHs) in gastric cancer cells oxidizing aldehydes into their corresponding carboxylic acids, as demonstrated by several authors [17,18]. If so, aldehydes present in the tissue, such as 3 methyl butanal, would be preferentially metabolized into carboxylic acids rather than into respective alcohols. This finding is in excellent agreement with the results of some in vitro studies. More specifically, the downregulated production of some primary alcohols such as 3-methyl-1-butanol was noted in several gastric cancer cell lines: SNU-1, AGS, HGC-27, and CLS-145 [19,20]. The same mechanism might be responsible for the downregulated production of ethyl acetate. The production of this compound involves the esterification reaction employing ethanol and acetic acid. The latter VOC is commonly produced in several human biochemical pathways (e.g., the Krebs cycle or by pyruvate metabolism). It may be also produced from ethanol by a tandem of ADHs and ALDHs. Thus, the production of this ester may indirectly reflect the activity of these enzymes. Their upregulated activity might reduce the ethanol levels in the tissue and thereby the ethyl acetate ones. Indeed, the total ADH activity has also been demonstrated to be significantly elevated in gastric cancer tissues [21].

Three ketones exhibited differences in the emission between the normal and cancer tissues. However, two of them (2-nonanone and cyclohexanone) showed upregulated production in cancer tissue, whereas the emission of 2-pentanone was found to be downregulated in this tissue. In principle, two metabolic pathways may be responsible for the production of ketones in humans: (i) oxidation of secondary or cyclic alcohols catalyzed by ADHs, or cytochrome p450 CYP2E1, and (ii) β-oxidation of fatty acids [16]. The former route is consistent with the aforementioned alterations in the cancer tissue metabolism and could thereby explain the elevated generation of cyclohexanone and 2-nonanal. If so, cyclohexanone would be the product of the cyclohexanol metabolism, an alcohol that has been detected in the tissues’ headspace. However, this pathway does not elucidate the lowered emission of 2-pentanone from gastric cancer tissues. Perhaps this ketone was produced in the alternative metabolic pathway, β-oxidation of fatty acids. 2-pentanone has been hypothesized to be formed via β-oxidation of hexanoic acid in the peroxisomal pathway [22]. Thus, the downregulation of one or more enzymes related to this route could lead to this alteration.

When it comes to aldehydes, several mechanisms could be responsible for their generation. These include (i) metabolism of alcohols by ADHs, (ii) lipid peroxidation, or (iii) diet. Production of C3–C10 aldehydes, such as n-hexanal or n-nonanal, was demonstrated to be associated with the reduction of hydroperoxides by cytochrome P450 (CYP450) through the lipid oxidation of omega-3 and omega-6 polyunsaturated fatty acids (PUFAs) [23]. Thus, n-hexanal observed in the Latvian population and n-nonanal found in the Ukrainian patients could be associated with secondary oxidative stress indicators. It is not clear why these VOCs did not exhibit analogous differences in both populations. Perhaps their very low concentration levels hindered the detection of the cancer-related alterations.

Interestingly, both populations under study exhibited contradictory alterations in isoprene emission from cancer tissues. Isoprene is a terpenoid emitted by humans in large quantities [24]. According to the current theory, in higher eukaryotes, isoprene is produced from isopentenyl pyrophosphate (IPP) and its isomer dimethylallyl pyrophosphate (DMAPP) in the mevalonic acid pathway [24]. Thus, isoprene is believed to be produced by acid-catalyzed formation from DMAPP occurring in the cytosol of hepatocytes. In humans, isoprene is metabolized in liver microsomes by cytochrome p450 (CYP2E1 and CYP2B6) to mono- and diepoxides (3,4-epoxy-3-methyl-1-butene and 3,4-epoxy-2-methyl-1-butene), which are in turn hydrolysed by epoxide hydrolase to vicinal diols (2-methyl-3-buten-1,2-diol and 3-methyl-3-buten-1,2-diol) [25].

γ-Butyrolactone occurs naturally in humans. However, it is also the metabolite of fluoropyrimidine (UFT), which is used in the treatment of different cancers, including gastric cancer [26].

Two heterocyclic compounds, pyrrole and pyridine, were found to be emitted in higher amounts by cancer tissue. Both VOCs are the components of numerous biologically active compounds. For instance, the pyrrole ring is a component of hemoglobin, myoglobin, and vitamin B12, and is produced during their biosynthesis [27]. The pyridine ring, in turn, builds nicotinic acid and nicotinamide, which are forms of vitamin B3 (niacin). Along with tryptophan, they are precursors to nicotinamide adenine dinucleotide (NAD^+^), nicotinamide adenine dinucleotide phosphate (NADP^+^), and their respective reduced forms (NAD(P)H). However, at this stage it is not clear what mechanisms underlie the upregulated emission of pyridine and pyrrole from cancer tissue. Perhaps these volatiles are the breakdown products of the aforementioned compounds. Further studies are required to elucidate the role of these VOCs in gastric cancer and reveal the underlying metabolic processes leading to their overproduction.

In the context of this study, the most interesting are metabolites exhibiting (i) high emission rates from the tissue samples, and at the same time (ii) the most notable difference in the emission between normal and cancer tissue samples in both populations. Only five compounds showed consistent alterations in both populations of patients. Amongst them, four volatiles, ethyl acetate, acetoin, 2,3-butanedione, and 3-methyl-1-butanol, were characterized by a reduced production in cancer tissue. A further compound, pyridine, was found to be overproduced by cancer tissue. From these observations, pyridine appears to have the highest potential as a volatile biomarker for gastric cancer. High emission rates and the significant change in its production induced by cancer render this species a very interesting candidate for a gastric cancer probe. Furthermore, pyridine overproduction by cancer tissue was also reported in our pilot study which also focused on gastric tissues [15]. Thus, the results of this study confirm this finding. However, it must be stressed that it is not clear if this upregulation is specific to gastric cancer. Further studies are required to clarify this issue.

## 5. Conclusions

The aim of this study was to characterize the volatile chemical patterns associated with cancer and normal tissues obtained from gastric cancer patients and identify potential volatile biomarkers of gastric cancer. The ex vivo GC-MS analysis resulted in the identification of 235 compounds, for which only 32% exhibited an incidence higher than 20%. Fourteen volatiles were found to be omnipresent (occurrence > 80%) in at least one type of tissue sample.

Nineteen volatiles showed consistent differences in their headspace concentrations above the samples under study. Headspace levels of eight species were significantly higher above the cancer tissue, whereas eleven VOCs were found to be elevated in the head space above the non-cancerous tissue. The difference in the emissions of the aforementioned species may be explained by cancer-related changes in the activity of certain enzymes or pathways.

Both populations investigated in this study also showed significant differences with respect to the cancer-related changes. Only one species, pyridine, exhibited elevated emission from cancer tissues in both populations of patients. Four species, pyrrole, phenol, cyclohexanol, and hexanal, were observed to be released in higher amounts by the cancer tissues only in the Latvian population, whereas nonanal and 2-nonanol showed analogous alteration only in the gastric tissues from the Ukrainian patients. When it comes to downregulated VOCs, four compounds, ethyl acetate, acetoin, 2,3-butanedione, and 3-methyl-1-butanol, exhibited this hallmark in both populations under study. Furthermore, seven volatiles, 2-pentanone, γ-butyrolactone, DL-limonene, benzaldehyde, 2-methyl-1-propanol, benzonitrile, and 3-methyl-butanal were found in only one group of subjects.

The results obtained from this study imply that the human volatilome exhibits high interpopulation variability. This can be the reason for the disparities found between the volatile biomarkers of gastric cancer identified in different studies. In other words, clinical studies involving only one local population may provide only “local” biomarkers. It is not simple to explain the reason for this variability. However, local factors such as patients’ exposure to environmental VOCs, diet, or local microbiota may play a significant role. From this, we can state that the identification of “global” bioindicators is one of the major challenges in the use of volatilomics. However, the involvements of various cohorts of patients from different geographic locations could help to identify and overcome this problem.

In the context of this study, volatiles showing similar cancer-related changes in both populations might be considered as candidate biomarkers for gastric cancer. From our results, this applies to five compounds, namely, pyridine, ethyl acetate, acetoin, 2,3-butanedione, and 3-methyl-1-butanol. Owing to the levels produced, pyridine appears to be the most promising of these compounds. Although the metabolic pathways leading to the production of pyridine in humans are unclear, the high emission rates and its overproduction by cancerous tissue render this species a very interesting candidate for further investigations in terms of its use as a probe for gastric cancer. These further studies are required to elucidate specifically the role of pyridine in gastric cancer and to reveal the underlying metabolic processes leading to its overproduction.

The results obtained from this study confirm that the chemical fingerprint formed by volatiles in gastric tissue is definitely altered by gastric cancer. The volatile components of this fingerprint secreted from humans through breath could serve as biomarkers or probes of gastric cancer. They could lead the way to the development of non-invasive breath tests for the diagnosis of this disease. More specifically, the identification of volatiles associated with gastric cancer is an important step for guiding the design and optimisation of novel sensor technologies toward the robust and sensitive detection of disease-specific volatile markers.

There are several limitations of this study. First, the available tissue samples were relatively small (∼100 mg), which must have affected the sensitivity of the applied analytical method, and thereby the recognition of species emitted in trace quantities. Secondly, the healthy tissue samples could be contaminated by the VOCs released by the malignant tissues, as the paired samples stemmed from the same stomach. Thus, the observed differences in emission may be diminished, affecting the identification of potential biomarkers.

## Figures and Tables

**Table 1:** Patients’ recruitment details from the two clinical centres involved (LOC and UNCL).

Clinical Centre	Number of Patients	Females	Males	Age Range (Median)
LOC	65	16	49	35–87 (66)
UNCI	29	8	21	47–78 (66)
Total	94	24	70	

**Table 2:** Retention times (Rt) (min), m/z of the quantifier ions, LODs (ppb), RSDs (%), coefficients of variation (R2), and linear ranges (ppb) for compounds of interest.

VOC	CAS	R_t_ [min]	Quantifier Ion m/z	LOD [ppb]	RSD [%]	R^2^	Linear Range [ppb]
2,3-Butanedione	431-03-8	6.236	43	0.06	9	0.998	0.18–42
Ethyl acetate	141-78-6	6.684	43	0.08	6	0.998	0.24–35
2-Methyl-1-propanol	78-83-1	9.500	74	1.09	19	0.990	3–30
Butanal, 3-methyl-	590-86-3	9.889	58	0.06	14	0.997	0.21–32
2-Pentanone	107-87-9	13.242	86	0.03	10	0.996	0.09–24
Pyridine	110-86-1	16.943	52	0.37	6	0.994	1.1–50
1-Butanol, 3-methyl-	123-51-3	17.952	70	0.11	6	0.989	0.33–29
Hexanal	66-25-1	20.584	57	0.14	5	0.992	0.42–26
Pyrrole	109-97-7	20.620	67	0.07	6	0.997	0.21–32
Cyclohexanol	108-93-0	25.289	57	0.03	7	0.992	0.09–26
Cyclohexanone	108-94-1	25.693	98	0.02	9	0.997	0.06–26
γ -Butyrolactone	96-48-0	28.400	86	0.23	9	0.997	0.69–40
DL-Limonene	5989-27-5	29.419	68	0.47	9	0.989	1.5–25
Benzonitrile	100-47-0	29.900	103	1.07	9	0.996	3–32
2-Nonanone	821-55-6	32.650	58	1.17	18	0.990	3–20

**Table 3:** Compounds exhibiting differences in emission between normal and cancer tissues.

		Total				LV *				UA **			
VOC	CAS	Change	Incidence [%]	*p*-Value ****	Median Fold Increase T/N ***	Change	Incidence [%]	*p*-Value	Median Fold Increase T/N	Change	Incidence [%]	*p*-Value	Median Fold Increase T/N
Benzaldehyde	100-52-7									↓ ^	93	0.03	0.94
1-Propanol, 2-methyl-	78-83-1									↓	52	8.5 × 10^−4^	0.3
2-Pentanone	107-87-9					↓	94	0.032	0.86				
Ethyl Acetate	141-78-6	↓	82	2.5 × 10^−5^	0.56	↓	91	2.3 × 10^−4^	0.7	↓	59	0.027	0.37
Benzonitrile	100-47-0									↓	48	0.017	0.1
Acetoin	513-86-0	↓	67	4.4 × 10^−4^	0.48	↓	67	2.6 × 10^−3^	0.42	↓	69	0.026	0.67
2,3-Butanedione	431-03-8	↓	67	6.5 × 10^−7^	0.72	↓	72	2.2 × 10^−5^	0.67	↓	55	4.4 × 10^−3^	0.46
γ-Butyrolactone	96-48-0					↓	54	6.1 × 10^−6^	0.62				
1-Butanol, 3-methyl-	123-51-3	↓	37	5.7 × 10^−6^	0.23	↓	28	3.6 × 10^−3^	0.33	↓	59	2.7 × 10^−4^	0.13
Butanal, 3-methyl-	590-86-3									↓	50	0.017	0.37
D-Limonene	5989-27-5					↓	38	0.02	0.67				
Pyridine	110-86-1	↑ ^^	81	1.9 × 10^−5^	2.6	↑	86	2.7 × 10^−4^	2.2	↑	69	6.5 × 10^−3^	6.3
Nonanal	124-19-6									↑	76	4.2 × 10^−3^	1.5
2-Nonanone	821-55-6									↑	72	0.027	1.6
Phenol	108-95-2					↑	62	4.4 × 10^−3^					
Pyrrole	109-97-7					↑	62	0.04	1.4				
Cyclohexanone	108-94-1					↑	68	3.7 × 10^−3^	1.3				
Hexanal	66-25-1					↑	51	7.3 × 10^−3^	1.3				
Isoprene	78-79-5					↓	57	4.4 × 10^−3^	0.6	↑	30	0.015	3.6

* LV—Latvian Population, ** UA—Ukrainian Population, *** T—cancerous tissue, N—healthy tissue, **** *p*-values refer to Wilcoxon signed rank test, ^ ↓—downregulated, ^^ ↑—upregulated.

**Table 4:** Concentrations of volatiles of interest over the tissue samples.

VOC	CAS	Headspace Concentration [ppb] Range (Median)					
		Total		LV *		UA **	
		N ***	T ***	N	T	N	T
Benzaldehyde	100-52-7	nq	nq	nq	nq	nq	nq
1-Propanol, 2-methyl-	78-83-1	–	–	–	–	1–746 (13)	1–655 (2.5)
2-Pentanone	107-87-9	–	–	0.1–26 (1)	0.1–40 (0.8)	–	–
Ethyl Acetate	141-78-6	0.24–115 (2.5)	0.24–50 (1.6)	0.24–40 (3)	0.24–33 (1.6)	0.24–115 (1.7)	0.24–50 (0.8)
Benzonitrile	100-47-0	–	–	–	–	1–4 (1.5)	1–3 (1)
Acetoin	513-86-0	nq	nq	nq	nq	nq	nq
2,3-Butanedione	431-03-8	0.18–290 (0.69)	0.18–147 (0.4)	0.18–16 (0.56)	0.18–9,5 (0.38)	0.18–290 (5.3)	0.18–147 (3.8)
γ -Butyrolactone	96-48-0	–	–	0.69–85 (11.2)	0.69–51 (0.23)	–	–
1-Butanol, 3-methyl-	123-51-3	0.1–3100 (5)	0.1–98 (0.5)	0.1–3 (0.6)	0.1–1.3 (0.2)	0.1–3100 (20)	0.1–98 (3.3)
Butanal, 3-methyl-	590-86-3	–	–	–	–	0.2–105 (4)	0.2–526 (3.5)
D-Limonene	5989-27-5	–	–	0.1–162 (1.5)	0.4–50 (1)	–	–
Pyridine	110-86-1	1.1–1870 (27)	1.1–7100 (87)	1.1–1870 (36)	1.1–2763 (79)	1.1–629 (5)	1.1–7100 (163)
Nonanal	124-19-6					1–7 (1.7)	1–11 (2.6)
2-Nonanone	821-55-6					1–50 (4)	1–57 (5)
Phenol	108-95-2	nq	nq	nq	nq	nq	nq
Pyrrole	109-97-7			0.4–32 (1.8)	0.4–68 (2.5)		
Cyclohexanone	108-94-1			0.06–95 (8.2)	0.09–162 (16)		
Hexanal	66-25-1			0.15–4 (0.56)	0.14–4.1 (1)		
Isoprene	78-79-5			0.2–6 (1.1)	0.2–2.23 (0.8)	0.2–3.2 (0.4)	0.8–66 (3.5)

* LV—Latvian Population, ** UA—Ukrainian Population, *** T—cancerous tissue, N—healthy tissue, nq—not quantified.

## Data Availability

The data presented in this study are available on request from the corresponding author.
